# Effects of Static Magnetic Field on Compression Properties of Mg-Al-Gd Alloys Containing Gd-Rich Ferromagnetic Phase

**DOI:** 10.3390/ma13214957

**Published:** 2020-11-04

**Authors:** Qi Cai, Xinyao Li, Shukui Li, Chuan He, Xingwei Liu, Xinya Feng

**Affiliations:** 1 School of Materials Science and Engineering, Beijing Institute of Technology, Beijing 100081, China; caiqi@shu.edu.cn (X.L.); bitleesk@bit.edu.cn (S.L.); chuan.he@bit.edu.cn (C.H.); xwliu@bit.edu.cn (X.L.); fengxinya@bit.edu.cn (X.F.); 2 China National Key Laboratory of Science and Technology on Materials under Shock and Impact, Beijing Institute of Technology, Beijing 100081, China; 3 State Key Laboratory of Explosion Science and Technology, Beijing Institute of Technology, Beijing 100081, China

**Keywords:** magnesium alloys, static magnetic field, cooling rate, second phase, mechanical properties

## Abstract

The Mg–0.6Al–20.8Gd (wt.%) alloys were homogenized at 620 °C for 20 min under 0 T and 1 T, followed by furnace cooling, quenching, and air cooling, respectively. The effects of the magnetic field on the phase constituent, microstructure, secondary phase precipitation, and mechanical properties of the Mg–Al–Gd alloys were investigated. The Mg–Al–Gd alloys contained α-Mg, Mg_5_Gd, Al_2_Gd, and GdH_2_ phases, and the phase constituents were hardly influenced by the applied magnetic field. However, the precipitation of the paramagnetic Mg_5_Gd upon cooling was accelerated by the magnetic field, and that of the ferromagnetic Al_2_Gd phases was inhibited. In addition, the Al_2_Gd phase was significantly refined and driven to segregate at the grain boundaries by the magnetic field, and the resultant pinning effect led to the microstructure change from dendritic α-Mg grains to rosette-like ones. When the magnetic field was only applied to the homogenization stage, the content of the Mg_5_Gd phase remained unchanged in the quenched alloy, whereas the Mg_5_Gd laths were significantly refined. By contrast, the contents of the Al_2_Gd and GdH_2_ phases were increased, while the precipitation sites were still within the α-Mg grains. The Mg_5_Gd laths were incapable of providing precipitation strengthening, while the Al_2_Gd and GdH_2_ particles brought positive effects on the enhancement of the mechanical properties. In the quenching condition, the hardness, compression strength, and ductility can be improved by the magnetic treatment, whereas these mechanical properties can be suppressed in the furnace cooled condition by the magnetic treatment.

## 1. Introduction

Magnesium alloys with low density have been the focus of worldwide attention due primarily to their potential for applications in the automotive industry. Taking the advantages of high specific strength, excellent shock resistance, and good damping capacity, the magnesium alloys have been used for fabricating the inner components of the vehicles to follow the strategies of weight reduction and energy conservation [1,2]. To overcome the problem of poor corrosion resistance for pure magnesium, rare earth (RE) elements were commonly added to the magnesium alloys, and the strength and thermal stability were also significantly improved [3,4,5,6]. So far, researchers still put the efforts on enhancing the mechanical properties of the Mg–RE alloys, with the purpose of expanding their application fields, e.g., in the car body and the automobile skeleton, where Al alloys are currently used. However, the true strength of the Mg alloys is uncompetitive, compared with that of the Al alloys.

Overall, the improvement of the mechanical properties has relied mainly on grain refinement and precipitation strengthening in Mg alloys. Based on the Mg-RE system, Al_2_RE particles were further introduced to refine the α-Mg grains for a combined enhancement of strength and ductility by adding a minor amount of Al element [7,8]. The Mg-Al-RE alloys may contain α-Mg phase, Mg-Al compounds, and Al-RE compounds. Zhang et al. found that the Al_2_RE phase hindered the dislocation climbing and grain boundary sliding for the enhanced strength of the Mg-4Al-RE alloys [9]. However, coarse intermetallic phases would result in low creep resistance and low mechanical properties [10]. Recently, Wei et al. reported that the Al_2_(Gd, RE) phase not only refined the α-Mg grains, but also modified the acicular Al_11_RE_3_ into a rod-like phase in the Gd-contained Mg-Al-RE alloys, leading to the enhancement of the mechanical properties [11]. For the Mg–Gd–Al–Zn alloys, Pourbahari et al. proved that the main strengthening phases were also the Gd- and Al-contained intermetallics, especially Al_2_Gd or (Mg,Al)_3_Gd. [12,13]. In view of this, the regulation of the Al-RE phase is the key to improve the comprehensive mechanical properties of the Mg–Al–RE alloys.

Amongst the RE elements, Gd has the highest solubility in the α-Mg phase, and the commercial Mg–Gd-based alloys exhibited excellent strength and creep resistance. The Mg–Al–Gd alloys have attracted great attention, since the long period stacking-ordered (LPSO) phase might form in the alloys [14,15,16]. However, studies of the Mg–Al–Gd alloys mostly focused on the characterization of the LPSO phase by high-resolution transmission electron microscopy. It is also essential to investigate the mechanical properties of the Mg–Al–Gd alloys by tuning the content and morphology of the Mg–Gd and Al–Gd secondary phases.

In contrast with hot extrusion and hot rolling, magnetic field treatment is a contamination-free route to regulate the microstructure of the ferromagnetic materials. Studies also showed that the magnetic field would modify the microstructure of the non-magnetic materials, if the magnetism of the materials was anisotropic [17]. For instance, a weak magnetic field (*B* ≤ 0.5 T) significantly affected the cellular liquid–solid interface and the cellular morphology in Al–Cu hypoeutectic alloys [18]. For the Mg–Zn–Y alloys, the magnetic field changed the orientation plane of the α-Mg phase, and the supposed refinement of the α-Mg grains was determined by the magnetic field intensity [19]. The phenomenon of the grain refinement was also observed in the Mg–Gd–Zn and Mg–Y alloys under the magnetic field, accompanied by the enhanced strength [20,21]. In addition to the refinement of the α-Mg grains, our previous study elucidated that the static magnetic field was able to refine the LPSO laths as well in the Mg–Al–Gd alloys [22]. However, the effects of the magnetic field on the content and morphology of Mg–Gd and Al–Gd secondary phases in the Mg–Al–Gd alloys remained undiscovered.

In this study, the effects of the magnetic field on the content, size, and shape of the secondary phases were investigated in the Mg–Al–Gd alloys. The function of the magnetic field was separately analyzed at the solution treatment stage and the cooling stage, respectively, by using different cooling rates. Quenching was used to freeze the phase constituent and microstructure of the alloy right after solution treatment. Air cooling was used to exclude the effect of the magnetic field upon cooling. Lastly, the effects of the magnetic field on the compression properties of the Mg–Al–Gd alloys were clarified.

## 2. Materials and Methods 

Ingots of Mg–0.6Al–20.8Gd (wt.%) ternary alloy was prepared by high frequency induction melting in Ar. The composition is measured by a P400 type inductively coupled plasma emission spectrometer (ICP, PerkinElmer, Waltham, MA, USA). The ingots were then cut into rectangular specimens with a dimension of 15 mm × 5 mm × 5 mm, and the specimens were homogenized at 620 °C for 20 min under the static magnetic field of 1 T, followed by quenching, air cooling, and furnace cooling, respectively. Specimens were also treated under the same heat treatment condition but without the magnetic field as a comparison. The solution treated alloys were characterized by X-ray diffraction (XRD, Bruker D8 Advance CuKα, Karlsruhe, Germany), optical microscopy (OM, Olympus BX41M, Tokyo, Japan), scanning electron microscopy (SEM, Hitachi S-4800, Tokyo, Japan), and transmission electron microscopy (TEM, JEM–2100, JEOL, Tokyo, Japan). Specimens for SEM observation were etched by 4.0 vol.% HNO_3_–ethanol solution, and those for TEM observations were electropolished in a solution of 1.5 vol. HClO_4_ and 98.5 vol.% ethanol under 40 V at −45 °C. The room temperature microhardness was measured by the Vickers hardness test (0.2 kg load) according to the ASTM E384-17 standard. The room temperature compression tests were performed on an electronic universal testing machine (MTS C45.305) at a compressive rate of 0.2 mm·min^−1^. The dimension of the compression specimens is Φ3 × 4.5 mm.

## 3. Results and Discussion

**Figure 1:**
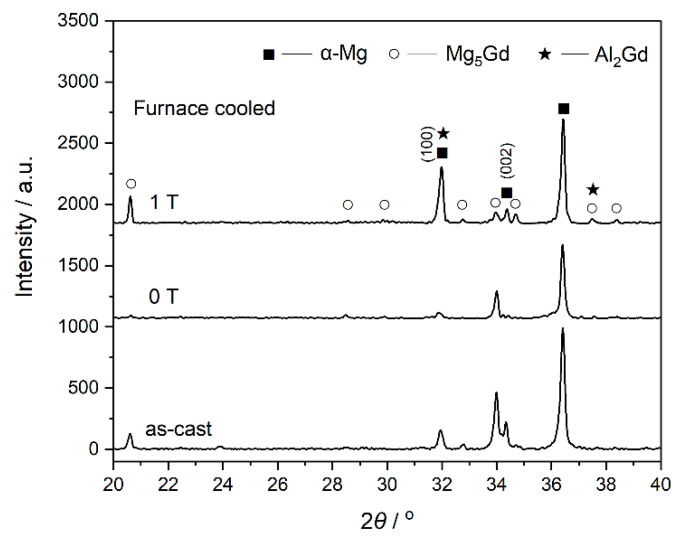
XRD patterns of the as-cast Mg–Al–Gd alloy and those homogenized at 620 °C and furnace-cooled under 0 T and 1 T.

**Figure 2:**
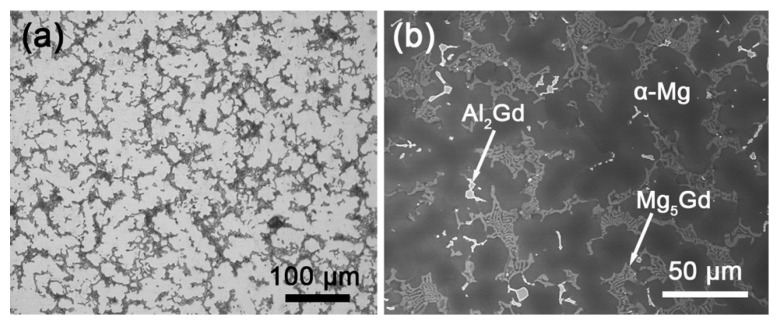
(**a**) OM and (**b**) SEM images of the as-cast Mg–Al–Gd alloy.

The XRD pattern of the as-cast Mg–Al–Gd alloy is shown in Figure 1. Combined with the OM and SEM observations (Figure 2), the as-cast Mg–Al–Gd alloy was composed of rosette-like α-Mg, eutectic Mg_5_Gd, and polygonal Al_2_Gd phases, while no LPSO phase was generated. The α-Mg+Mg_5_Gd eutectic structure was distributed among the α-Mg grains, and the Al_2_Gd particles with tales located within the α-Mg grains. The shape and dimension of the Al_2_Gd particles are similar to those in the Gd-contained AZ31 alloy [23], but these large particles might be unhelpful to the precipitation strengthening. Since the Al_2_Gd is a ferromagnetic C15-type Laves phase [24,25], it is expected that the magnetic field could be applied to tune the morphology of the Al_2_Gd particles for precipitation strengthening, with the help of homogenization and cooling. In view of this, the Mg–Al–Gd alloys were homogenized at 620 °C under 0 T and 1 T, respectively, to investigate the effect of the magnetic field on the phase constituent, microstructure, hardness, compression strength, and ductility. 

**Figure 3:**
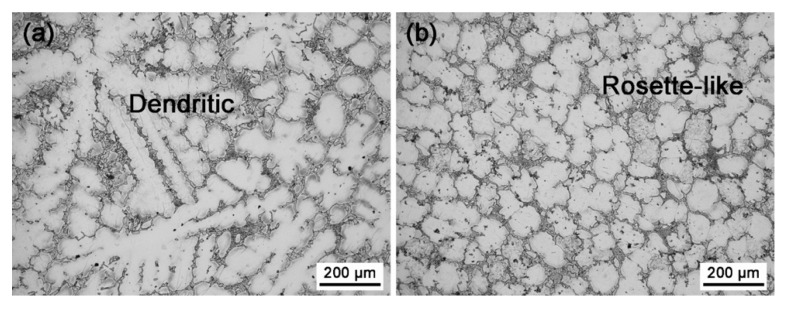
OM images of the Mg–Al–Gd alloys homogenized at 620 °C and furnace-cooled under (**a**) 0 T and (**b**) 1 T.

The XRD patterns of the furnace-cooled alloys under 0 T and 1 T are shown in Figure 1. Both the alloys homogenized with and without the magnetic field contained α-Mg, Mg_5_Gd, and Al_2_Gd phases. The phase constituent was the same as that of the as-cast alloy. Nevertheless, the phase content was affected by the magnetic field, in view of the change of the peak intensity in the XRD patterns. According to the Rietveld refinement method, the weight percentage of the phases was calculated [26], and the values are shown in Table 1. Homogenization under 0 T led to the dissolution of the Mg_5_Gd phase, and the content of Mg_5_Gd was reduced from 24.23 wt.% to only 4.10 wt.% in 20 min. When the homogenization was under 1 T, 12.17 wt.% Mg_5_Gd remained in the furnace cooled alloy, indicating that the applied magnetic field might have inhibited the dissolution of Mg_5_Gd. On the other hand, the orientation of the α-Mg phase was different in the alloys treated under 0 T and 1 T. The peak intensity of the (100)_α-Mg_ became significantly higher than that of the (002)_α-Mg_ in the alloy treated under 1 T. Although the α-Mg is a paramagnetic phase, the anisotropy of the magnetic susceptibility for the α-Mg phase, i.e., *χ*_(100)_ > *χ*_(002)_, resulted in such orientation [27]. Accompanied by the orientation of the α-Mg grains, the morphology of the α-Mg grains was changed by the applied magnetic field, as shown in Figure 3. Dendritic α-Mg grains were found in the alloy furnace cooled under 0 T (Figure 3a), while rosette-like grains were in the alloy furnace cooled under 1 T (Figure 3b). The length of the dendritic grains was up to 860 μm, and that of the rosette-like grains were only in 177 ± 18 μm. Compared with the dendritic grains, the rosette-like ones were significantly refined by the magnetic treatment. The magnetization force could be expressed as ***F***=(*χ*/*μ*_0_)(***B***·∇)***B***, where *χ* is the magnetic susceptibility, *μ*_0_ is the permeability of vacuum, ***B*** is the magnetic induction intensity, and ∇ is the gradient operator. The magnetic susceptibility of the paramagnetic materials is about 10^−3^, while that of the ferromagnetic materials is 10^3^. Therefore, the magnetization force in the paramagnetic phase could be neglected [17], and the paramagnetic α-Mg or Mg_5_Gd phase in the Mg–Al–Gd alloy was hardly modified by the magnetic force. Instead, the change in the shape of the α-Mg might be attributed to the ferromagnetic phase. It was also recognized that the grain size ranged from 150 μm to 860 μm in the alloy treated under 0 T, while the grains in the alloy treated under 1 T were uniform, indicating that the magnetic treatment is conducive to the homogeneous microstructure of Mg–Al–Gd alloys.

**Figure 4:**
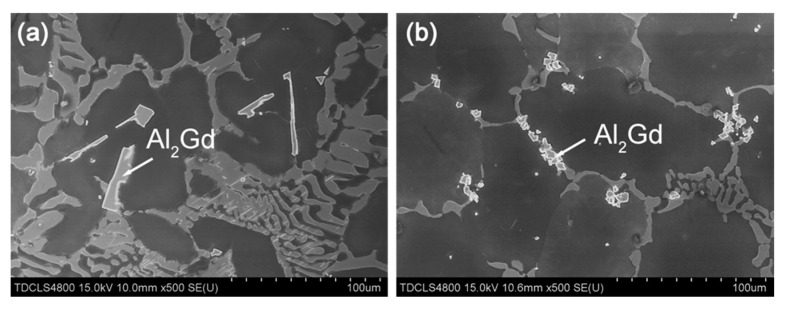
SEM images of the Mg–Al–Gd alloys homogenized at 620 °C and furnace-cooled under (**a**) 0 T and (**b**) 1 T.

In addition to the α-Mg phase, the morphology of the second phases was also impacted by the magnetic field. The SEM images of the Mg–Al–Gd alloys treated under 0 T and 1 T are shown in Figure 4. Although the content of the Mg_5_Gd phase at the grain boundaries was reduced in the furnace-cooled alloy under 1 T, the refinement of the α-Mg grains brought about an increasing number of grain boundaries, and the total content of the Mg_5_Gd phase in the alloy treated under 1 T was increased, which agreed well with the calculation results of the phase content (Table 1). Hu et al. have demonstrated that the mutual diffusion of Mg and Gd was accelerated by the magnetic field [28] for abundant dissolution, which would depress the segregation of Gd atoms. The homogeneous distribution of Gd atoms was the reason for the homogeneous precipitation of the Mg_5_Gd phase upon cooling. However, the magnetic field hardly affected the size of the eutectic Mg_5_Gd phase, and the width of the Mg_5_Gd laths was 5–12 μm in both the alloys were furnace cooled under 0 T and 1 T. Regarding the Al_2_Gd phase, the size of the Al_2_Gd particles was significantly decreased to be less than 10 μm by the magnetic field, and furthermore, the Al_2_Gd particles segregated at the grain boundaries. Since the Al_2_Gd is a ferromagnetic phase, the applied magnetic field will provide a driving force for its nucleation [29]. The increasing number of Al_2_Gd nuclei would be the reason for the refinement of the Al_2_Gd particles. Furthermore, the pinning effect of the fine secondary phase at the grain boundaries should be responsible for the grain refinement of the α-Mg [30]. 

**Figure 5:**
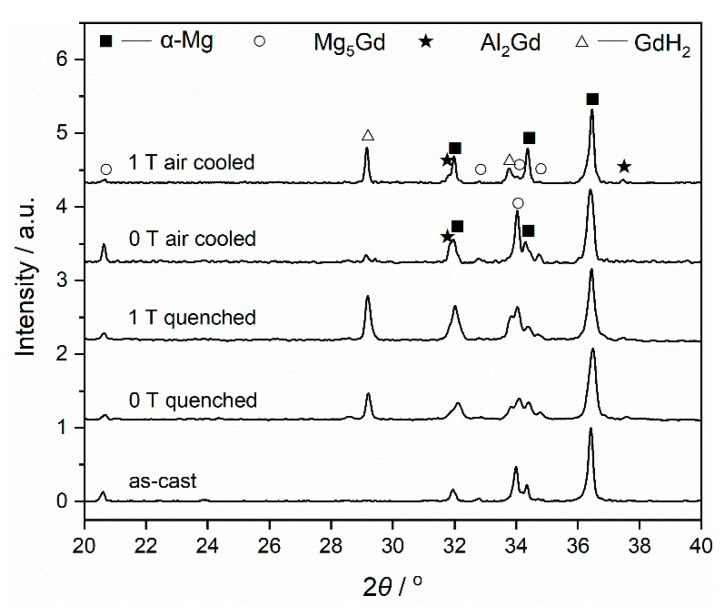
XRD patterns of the as-cast Mg–Al–Gd alloy and those quenched and air cooled under 0 T and 1 T.

**Figure 6:**
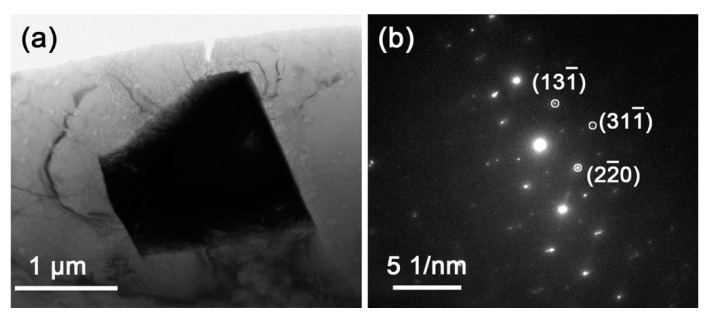
(**a**) TEM image of the Mg–Al–Gd alloy quenched under 1 T, and (**b**) selected area electron diffraction (SAED) pattern of the black particle in (a).

To figure out the effect of the magnetic field on the formation of the secondary phases upon cooling, quenching was used to freeze the high-temperature phase constituent and microstructure, and air cooling was used to exhibit the intermediate state between quenching and furnace cooling. The magnetic field was applied only at the homogenization stage, and the quenching and air cooling were under 0 T. Figure 5 shows the XRD patterns of the Mg–Al–Gd alloys quenched and air-cooled under 0 T and 1 T. A comparison was firstly made between the quenched Mg–Al–Gd samples to reveal the function of the magnetic field during the solution treatment. In addition to the α-Mg, Mg_5_Gd, and Al_2_Gd phases, both the quenched samples contained GdH_2_ hydride, which is commonly observed in the Mg-RE alloy [31]. The rare earth element may absorb atomic hydrogen to form a hydride in the water medium during quenching [32], and the formed GdH_2_ particles were reported to be cuboid or rectangle-shaped [31]. The formation of the GdH_2_ was also relative to the formation of the Mg_5_Gd upon cooling. It was the decomposition of the MgGdH_x_ into Mg_5_Gd and GdH_2_ [33], and the coherent orientation relationship is (111) Mg_5_Gd // (111) GdH_2_ [31]. Therefore, the content of GdH_2_ was relative to that of Mg_5_Gd. For the quenched and air-cooled alloy at 1 T, the content of Mg_5_Gd was 9.41 wt.% and 8.69 wt.%, respectively. Correspondingly, the content of GdH_2_ was 9.56 wt.% and 9.01 wt.%, respectively. The TEM image of the quenched Mg–Al–Gd alloy under 1 T was shown in Figure 6a to distinguish the GdH_2_ and Al_2_Gd phase. According to the selected area electron diffraction (SAED) pattern, the particle in Figure 6a was identified to be the GdH_2_ phase. The size of the GdH_2_ was about 1 μm, much smaller than that of the Al_2_Gd phase (~10 μm). The Al_2_Gd particles have been confirmed to have positive effects on the enhancement of the strength of the Mg–Al–Gd alloys [22].

**Figure 7:**
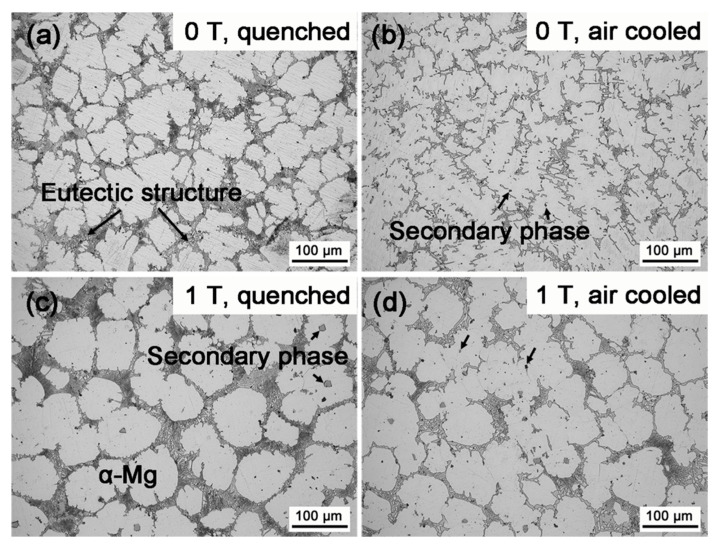
OM images of the quenched Mg–Al–Gd alloy under (**a**) 0 T and (**b**) 1 T, and the air cooled alloy under (**c**) 0 T and (**d**) 1 T.

**Figure 8:**
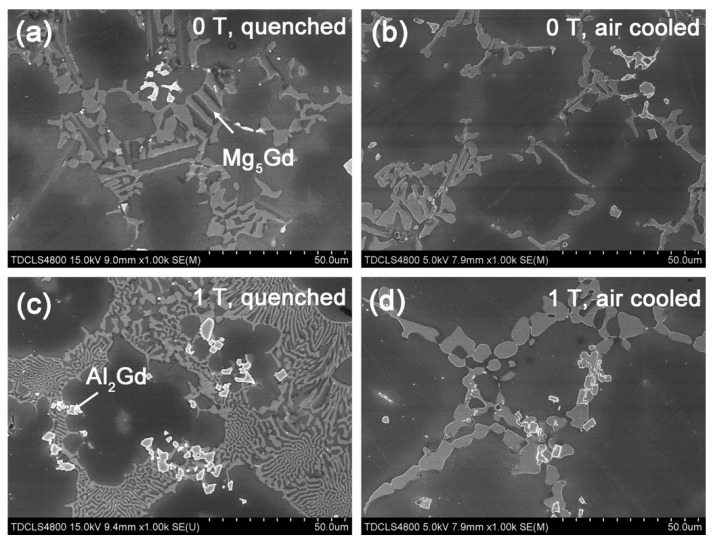
SEM images of the (**a**) quenched and (**b**) air-cooled Mg–Al–Gd alloys under 0 T, and (c) 1 T, and the air-cooled alloys under (**c**) 0 T and (**d**) 1 T.

During the homogenization, the magnetic field hardly changed the dissolution content of Mg_5_Gd, as the calculated weight percentage of Mg_5_Gd phase remained almost unchanged (Table 1). The orientation of the α-Mg phase in both the quenched alloys treated under 0 T and 1 T also remained the same as that of the as-cast alloy. Figure 7a,c shows the OM images of the quenched Mg–Al–Gd alloys after homogenization under 0 T and 1 T. Analogous to the as-cast alloy, both the quenched alloys contained the rosette-like α-Mg grains. The average size of the α-Mg grains was 81 ± 12 μm and 83 ± 14 μm for the quenched alloys treated under 0 T and 1 T, respectively. These phenomena indicated that the magnetic field brought about a minor impact on the phase constituent, content, orientation, and morphology of the paramagnetic phases during the solution treatment stage. It is then suggested that the applied magnetic field affected the morphology of the α-Mg and Mg_5_Gd upon cooling, rather than during the homogenization stage. Since the Mg_5_Gd content in the alloy furnace cooled under 1 T was almost three times larger than that in the alloy treated under 0 T, it was believed that the magnetic field accelerated the re-precipitation of Mg_5_Gd upon the furnace cooling stage, rather than inhibiting the dissolution of Mg_5_Gd during the homogenization stage. Regarding the ferromagnetic Al_2_Gd phase, the precipitation sites of the Al_2_Gd particles were examined. Figure 8a,c shows the SEM images of the quenched Mg–Al–Gd alloys after homogenization under 0 T and 1 T. The precipitation sites of the ferromagnetic Al_2_Gd phase were modified from the inner grains to the grain boundaries, when the magnetic field was applied. From the OM images, it was recognized that the second phase was still in the center of the α-Mg grains for the quenched alloy after homogenization under 1 T, as indicated by arrows (Figure 7c), while no secondary phases were found in the quenched alloy after homogenized under 0 T (Figure 7a). On the one hand, it was revealed that the Al_2_Gd particles could be the nucleation sites of the α-Mg, resulting in grain refinement of the α-Mg grains. On the other hand, this indicated that the precipitation of the Al_2_Gd phase was initiated during the homogenization process, and it eventually segregated at the grain boundaries upon the furnace cooling. Therefore, the Al_2_Gd particles restricted the growth of α-Mg grains throughout the homogenization and cooling stages. For the paramagnetic Mg_5_Gd phase, the homogenization under 1 T led to the significant refinement of the Mg_5_Gd laths, although the phase content was hardly changed. This originated from the mutual diffusion of Mg and Gd during the solution treatment [28], which led to the homogeneous precipitation of the Mg_5_Gd phase.

Air cooling was then used to exclude the effect of the magnetic field upon cooling. The XRD patterns of the air-cooled Mg-Al-Gd alloys after homogenization under 0 T and 1 T are shown in Figure 5. Since the magnetization force is too weak to bring visible impacts on the paramagnetic phase [17] within 20 min, one can hardly recognize the crystal orientation of either the α-Mg or the Mg_5_Gd from the XRD patterns of the quenched and air-cooled alloys (Figure 5). The air-cooled alloys were composed of α-Mg, Mg_5_Gd, Al_2_Gd, and GdH_2_, and the weight percentage of these phases is shown in Table 1. The phase constituent of the air-cooled alloy after homogenization under 0 T was close to that of the as-cast one, whereas the α-Mg grains began to reveal the feature of the dendrite (Figure 7b). When the alloy treated under 1 T was air-cooled, the α-Mg grains remained to be rosette-like (Figure 7d). This also proved that the solution treatment under the magnetic field has resulted in the refinement of the α-Mg grains. 

**Figure 9:**
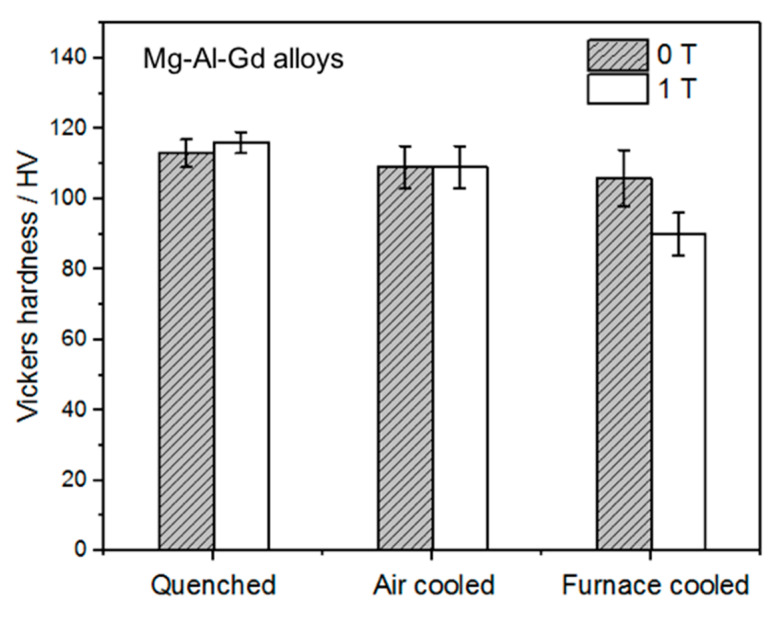
Vickers hardness of the quenched, air-cooled and furnace-cooled Mg–Al–Gd alloys under 0 T and 1 T.

The modification of the α-Mg and the secondary phases under the magnetic field will lead to the variation of the hardness and the compression properties. The Vickers hardness of the quenched, air-cooled, and furnace-cooled Mg–Al–Gd alloys under 0 T and 1 T is shown in Figure 9. Under different cooling conditions, the effect of the magnetic field on the hardness was different. To be specific, the hardness of the furnace-cooled alloy under 1 T was smaller than that of the alloy treated under 0 T, whereas magnetic treatment increased the hardness of the quenched alloy. As for the air-cooled alloys, the hardness was hardly influenced by the magnetic field. Sahoo et al. have clarified that the volume fraction of basal grains/orientations, which was reflected from the XRD patterns, decided the hardness of the Mg alloys [34]. The deviation from the (002) orientation would lead to a decrease in the hardness. Since the furnace-cooled alloy under 1 T contained a significantly increasing number of grains with the (100) orientation, it exhibited lower hardness than the alloy treated under 0 T. From another viewpoint, the magnetic field accelerated the precipitation of Mg_5_Gd at the grain boundaries, while in return the dissolved Gd atoms in the α-Mg grains were consumed, leading to the suppression of the solid solution hardening. Meanwhile, the precipitation of Mg_5_Gd with a negligible increase in dimension would lead to the decrease in the hardness [35]. These also accounted for the decreased hardness in the alloy treated under 1 T. 

**Figure 10:**
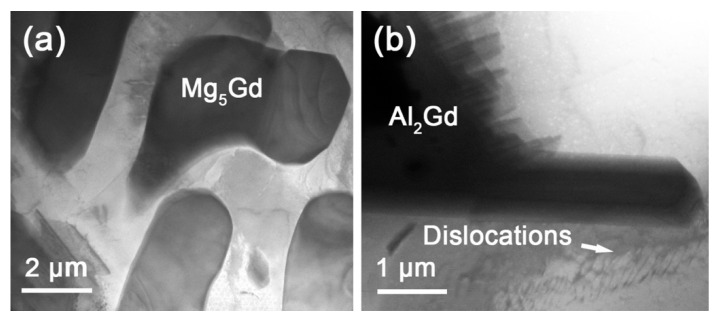
TEM images of the (**a**) Mg_5_Gd and (**b**) Al_2_Gd particles in the quenched Mg–Al–Gd alloys under 1 T.

When the magnetic field was applied, no orientation was identified from the XRD patterns for the quenched or air-cooled alloys. For the quenched alloys under 0 T and 1 T, the content of the Mg_5_Gd phase and the diameter of the α-Mg grains remained unchanged, while the contents of the Al_2_Gd and GdH_2_ phases were increased (Table 1). Also, part of the Al_2_Gd and GdH_2_ particles were still located within the α-Mg grains (Figure 8c) to provide precipitation hardening. The Al_2_Gd and GdH_2_ phases have high hardness, and could effectively pin dislocations for high hardness and strength [22,36]. Figure 10 shows the TEM images of the quenched Mg–Al–Gd alloy under 1 T. Direct observation of the pinning effects of Mg_5_Gd and Al_2_Gd for dislocations was achieved. The thick Mg_5_Gd laths were incapable of pinning the dislocations (Figure 10a), since no dislocations were observed around the Mg_5_Gd laths. On the contrary, ladder-like dislocation arrangements existed parallel to the side surface of the Al_2_Gd particle. Hence, the increased hardness for the Mg–Al–Gd alloy under 1 T may be attributed to the increasing Al_2_Gd and GdH_2_ contents (Table 1). For the air-cooled alloys, the reduced Mg_5_Gd phase brought positive effects on the enhancement of the hardness, which compensated the negative effects due to the growth of the α-Mg grains (Figure 7b,d). The alloys treated under 0 T and 1 T exhibited the same hardness of 109 ± 6 HV.

**Figure 11:**
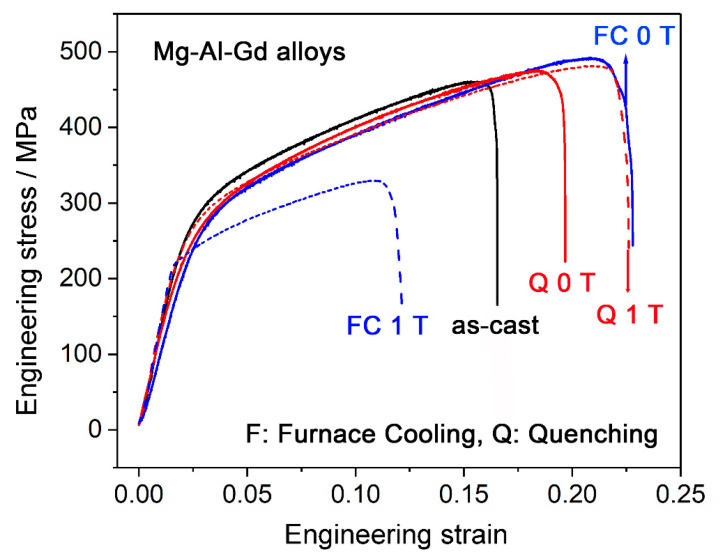
Compressive stress–strain curves of the as-cast Mg–Al–Gd alloy and the furnace-cooled and quenched alloys under 0 T and 1 T.

The compressive stress–strain curves of the quenched and furnace-cooled Mg–Al–Gd alloys under 0 T and 1 T are shown in Figure 11. The ultimate compression strength of the as-cast alloy was 460 MPa, and the strain to failure, *ε*_f_, was 0.16. Both the strength and ductility were improved by homogenization under 0 T followed by quenching and furnace cooling. The alloy furnace cooled under 0 T exhibited the highest ultimate strength of 490 MPa and the highest *ε*_f_ of 0.23. When the magnetic field was applied, the strength and ductility were decreased for the furnace-cooled alloy, in contrast with that of the alloy treated under 0 T. The furnace-cooled alloy under 1 T showed significantly reduced strength and *ε*_f_ of 329 MPa and 0.12, respectively. This tendency was similar to the variation of the hardness for the furnace-cooled alloys under 0 T and 1 T. As mentioned above, the increasing content of Mg_5_Gd and the reduced solid solution strengthening should be responsible for the depression of the compression strength. Moreover, the segregated Al_2_Gd particles at the grain boundaries would act as the source of cracks, and they would decrease the strength and ductility of the Mg–Al–Gd alloys. On the contrary, the quenched alloy under 1 T exhibited higher strength and ductility than the one treated under 0 T, and this tendency is also similar to the magnetic field-induced variation tendency of the hardness for the quenched alloys under 0 T and 1 T. The compression strength and *ε*_f_ of the quenched alloy under 1 T were 481 MPa and 0.22, respectively, which were comparable to those of the alloy furnace cooled under 0 T. Therefore, magnetic field treatment could enhance the strength and ductility of the Mg–Al–Gd alloy, combined with quenching. As mentioned above, the enhancement of the strength was due to the increasing amount of Al_2_Gd and GdH_2_ phase, which led to the entanglement of the dislocations at the interfaces (Figure 10). The refinement of Mg_5_Gd at the grain boundaries (Figure 8c) could account for the increase of the *ε*_f_.

## 4. Conclusions

The effects of the magnetic field on the content and morphology of the paramagnetic α-Mg, Mg_5_Gd, and the ferromagnetic Al_2_Gd phases were investigated in the Mg–0.6Al–20.8Gd alloys, as well as the mechanical properties of the alloys. The conclusions are drawn as follows.

(1) By comparing the furnace-cooled alloys under 0 T and 1 T, it was found that the magnetic field resulted in the orientation of the (100) crystal faces instead of the (200) faces for the α-Mg grains, which led to the decrease of the hardness for the furnace-cooled alloy. The magnetic field accelerated the precipitation of the Mg_5_Gd phase upon cooling, and in return inhibited the precipitation of the Al_2_Gd phase. Although the Al_2_Gd particles were refined, the remained Al_2_Gd particles were driven to precipitate at the grain boundaries of the α-Mg phase by the magnetic field. Therefore, the precipitation strengthening was suppressed, and the mechanical properties of the furnace-cooled Mg–Al–Gd alloys under the magnetic field were decreased.
(2) For the quenched alloys after homogenization under the magnetic field, no orientation of the α-Mg phase was identified. Although the content of the Mg_5_Gd phase was hardly changed in the alloys, the eutectic Mg_5_Gd laths were significantly refined by the magnetic field. In addition, the contents of the Al_2_Gd and GdH_2_ strengthening phases were increased in the alloy treated under 1 T, and the Al_2_Gd and GdH_2_ particles were still located within the α-Mg grains. Therefore, the hardness, compression strength, and ductility of the Mg–Al–Gd alloys were improved under the magnetic field, in contrast with the as-cast alloy, as well as the one without magnetic treatment.

## Figures and Tables

**Table 1:** Weight percentage of the Mg–Al–Gd alloys homogenized under 0 T and 1 T, followed by furnace cooling, quenching, and air cooling.

		Magnetic Field/T	Phase Content/wt.%			
			α-Mg	Mg_5_Gd	Al_2_Gd	GdH_2_
**As-cast**	**N/A**	N/A	73.26	24.23	2.51	/
620 °C, 20 min	Furnace cooled	0	93.02	4.10	2.19	/
		1	85.28	12.17	1.44	/
	Quenched	0	82.82	9.34	0.77	7.07
		1	79.82	9.41	1.21	9.56
	Air cooled	0	73.75	23.16	1.27	1.83
		1	79.35	8.69	2.96	9.01
